# Supplementary material for: Immune response to the mRNA COVID-19 vaccine in hemodialysis patients: cohort study
Source: medRxiv. 2023 Jan 19:2023.01.19.23284792. Preprint. [Version 1] doi: 10.1101/2023.01.19.23284792 (PMC9882629; doi:10.1101/2023.01.19.23284792)
Supplement: Supplement 1 [file NIHPP2023.01.19.23284792v1-supplement-1.pdf]

medRxiv preprint doi: https://doi.org/10.1101/2023.01.19.23284792; this version posted January 19, 2023. The copyright holder for this preprint (which was not certified by peer review) is the author/funder, who has granted medRxiv a license to display the preprint in perpetuity.
It is made available under a CC-BY 4.0 International license .

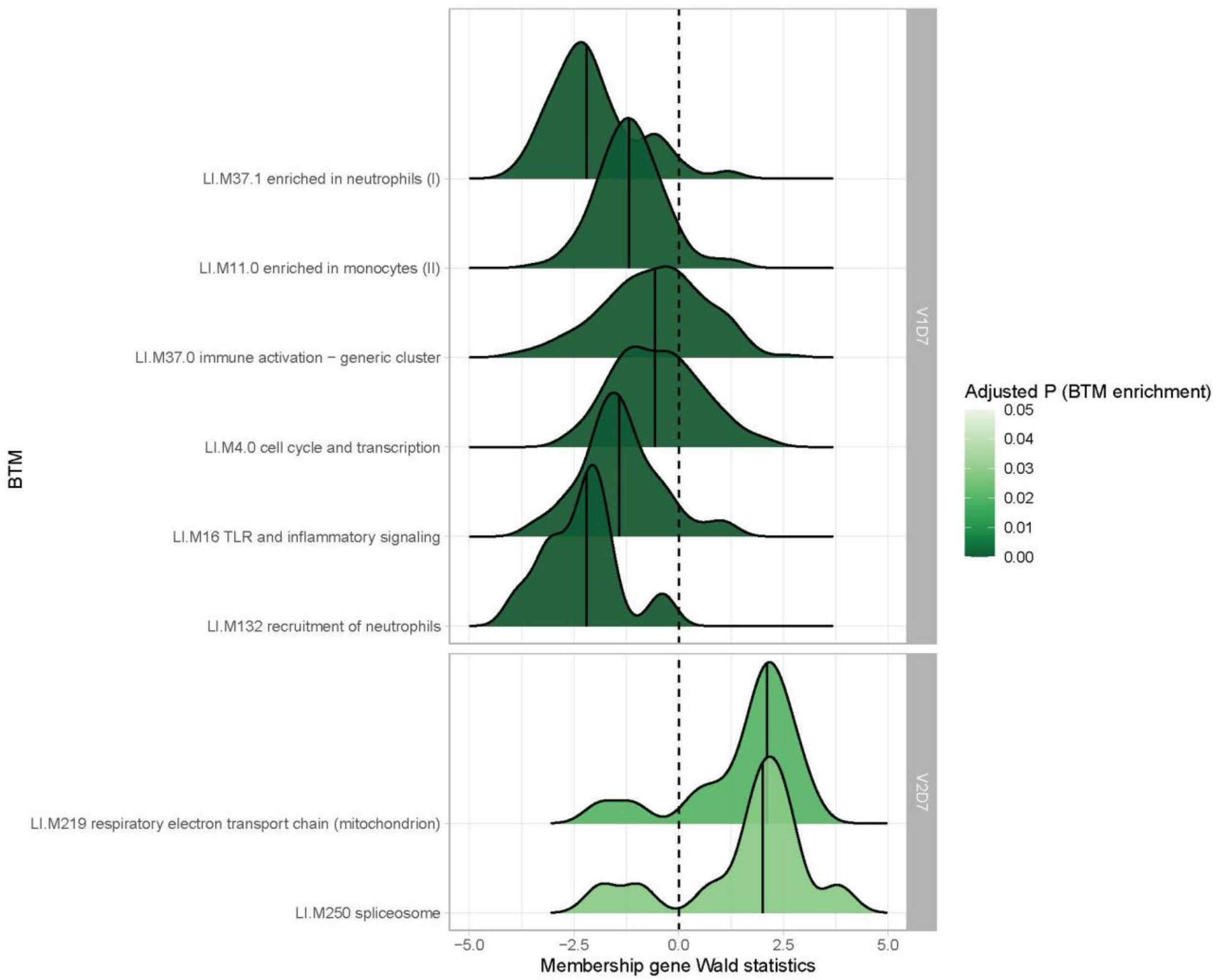

medRxiv preprint doi: https://doi.org/10.1101/2023.01.19.23284792; this version posted January 19, 2023. The copyright holder for this preprint (which was not certified by peer review) is the author/funder, who has granted medRxiv a license to display the preprint in perpetuity.
It is made available under a CC-BY 4.0 International license .

Figure S1. Hemodialysis patients (HD) without prior SARS-CoV-2 infection show increased myeloid activity at V1D7 and decreased metabolic activity at V2D7 compared to controls (HC). The most differentially enriched blood transcription modules (BTMs) between HC and HD with no prior infection with SARS-CoV-2 are shown ($p < 0.05$, FDR-adjusted) at V1D7 and at one week after second vaccination dose (V2D7). Density plots for each BTM represent Wald statistics from DESeq2 analysis for each membership gene per BTM, with positive Wald statistics indicating increased expression in HC compared to HD.

medRxiv preprint doi: https://doi.org/10.1101/2023.01.19.23284792; this version posted January 19, 2023. The copyright holder for this preprint (which was not certified by peer review) is the author/funder, who has granted medRxiv a license to display the preprint in perpetuity.
It is made available under a CC-BY 4.0 International license .

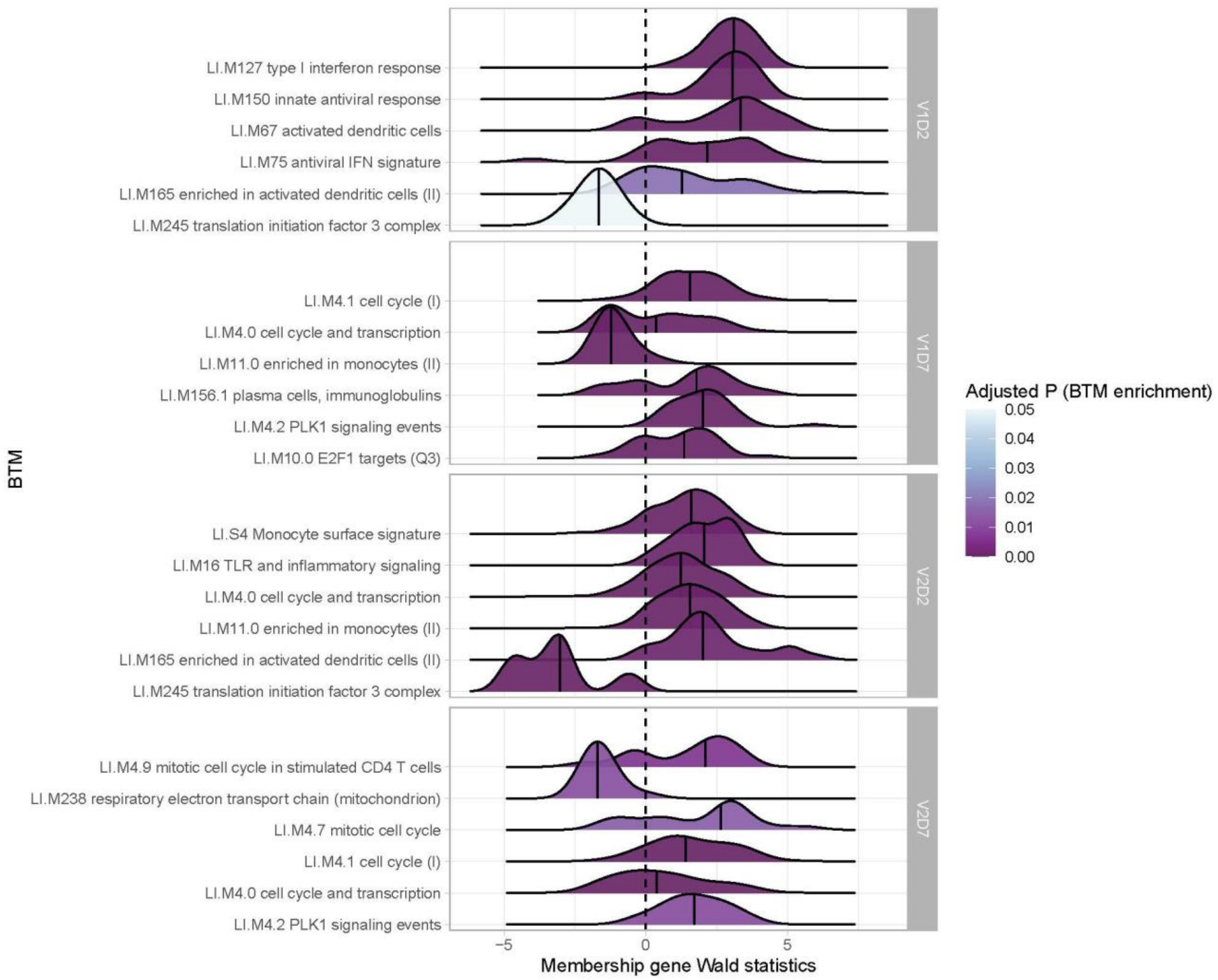

Figure S2. Hemodialysis patients (HD) with prior SARS-CoV-2 infection show increased expression of innate and adaptive immune blood transcription modules (BTMs) post-vaccination. The most significantly enriched BTMs are shown (up to six) for Day 2 (D2) and Day 7 (D7) after each vaccination dose (V1, V2) in HD with prior infection with SARS-CoV-2 ($p < 0.05$, FDR-adjusted). Density plots for each BTM represent Wald statistics from DESeq2 analysis for each membership gene, thereby representing increased or decreased expression per gene at each time point compared to baseline (V1D0 or V2D0).
